# Contributions to Pathological Phrenology

**Published:** 1833-03

**Authors:** H. H. Holm

**Affiliations:** Lecturer on Phrenology in the School of Medicine, London Hospital.


### PATHOLOGICAL PHRENOLOGY.

*Contributions to Pathological Phrenology.* By H. H. Holm, Esq., Lecturer on Phrenology in the School of Medicine, London Hospital.

Case I. On Tuesday, the 18th of August, 1832, I visited — Horsey, aged about thirty-four. On entering the room, I remarked extreme excitement of the whole system, so much so, that all the muscles appeared to be slightly convulsed: he was not entirely incoherent, but laboured evidently under delusions, the purport of which seemed to be, that there were disturbances which either annoyed him or in which he considered it necessary to implicate himself. On examining the head, I was struck with the greater heat about the *region of circumspection*, namely, near the point of ossification of the parietal bones, which extended in a circle about four inches in diameter. This appeared very obvious to the two medical gentlemen who, besides myself, were present. We endeavoured to quiet him by kind conversation, and we agreed that the hair should be shaved off the heated region; six leeches applied on either side, (he being previously undressed, and reclining on the bed, with a view to his getting sleep;) and we ordered that afterwards the room should be kept dark and free from noise. Aloetic purgatives were prescribed, with a view to act on the lower bowels; and light diet, with a glass of port wine, allowed, he having been addicted to drinking.

*Previous history.* Was porter to the Royal College of Surgeons in London, and had been discharged for impertinence to one of the officers, who exercised, as Horsey thought, too great *military* authority. After his discharge, (or, according to his own statement, after he had sent in his resignation, on the plea of ill health, on which account his wages were continued for a month, during his stay in the country,) he went to his friends; but, not meeting with employment that suited him, he returned to town. For many days before his attack, he had been drinking large quantities of brandy and other spirits. He complained of his inability to sleep.

After a second application of the leeches, at an interval of two days, he gradually became more rational, and, by raising his hopes of being reinstated in his former situation, he was eventually restored to health.

In tracing the immediate causes of excitement, a due weight should be attached to the effect of the spirits he took; but they merely excited into more violent action those feelings of caution previously over active: and this case combines the partial disease and the inflammation of a *particular* portion of the brain.

Case II. October 1832. J. B., about two months ago, during a violent thunder-storm, was standing at the garden door, when a flash of lightning so terrified him, that he never appeared to recover from the fright. His father observed that the expression of his countenance changed, and never recovered its natural appearance, from that time to his death. His mother said that, from the period referred to, he became fractious, exceedingly timid, and had lost all the quickness he had previously manifested.

He was treated by his medical attendants for inflammation of the brain; but it appears that the symptoms were never effectually subdued. About five weeks before his death, (i. e. three weeks after the fright,) he was seized with a convulsive fit, which lasted five hours, and which was followed by hemiplegia; and, although the paralysis was relieved, he never completely recovered the use of the affected side.

On Friday last he became completely senseless, and died yesterday (i. e. Sunday) at twelve o'clock.

*Autopsy.* On stripping off the pericranium, there were seen distinct red lines along the course of the frontal and sagittal sutures; and the same appearance was noticed on the inner side of the bones, when the calvarium was removed. Serum was found effused between the membranes; and the vessels of the pia mater were more than usually filled, exhibiting the general appearances of inflammation. The convolutions of the anterior lobes were very soft, more particularly those lying on the central parts of the orbital plates of the frontal bones; so that, on lifting them up to divide the nerves at the base, the fingers penetrated them. The corpora striata, or great anterior ganglions, from which emerge the anterior convolutions, were also exceedingly soft; while the posterior ganglions, or optic thalami, and the convolutions proceeding from them, were of the natural consistence.

It should also be observed, that the child had, by continual scratching, produced a small wound at the lower part of the forehead, in the mesial line, where the convolutions were found most soft and altered in texture. (Query, was this the effect of the pain occasioned internally?)

Now, although on many children the fright would hardly be sufficient to account for the cause of so much mischief, it will appear more evident on relating the predisposing circumstances. The parents had previously lost a child from acute inflammation of the brain, about the same age (two years and a half), a period of life at which this disease is very frequent. The temperament of this boy was nervo-sanguine, and he shewed great cerebral activity. Before the immediate cause of this attack, he had been visiting at an aunt's, where he had been indulged, and the functions of the stomach had become deranged, so that the mother even suspected he had received some injury while from home.

This case appears to be in consonance with the physiology and structure of the brain, as shown by Dr. Spurzheim, and which affords the only satisfactory means of explaining the direct effects of the injury sustained, viz. loss of intellectual functions, combined with immediate injury to the convolutions belonging to the intellectual operations.

